# Association of Surrogate Objective Measures With Work Relative Value Units

**DOI:** 10.31486/toj.20.0153

**Published:** 2021

**Authors:** Tam Ramsey, Tyler Ostrowski, Kent Curran, Jason Mouzakes, Neil Gildener-Leapman

**Affiliations:** Department of Otolaryngology–Head and Neck Surgery, Albany Medical Center, Albany, NY

**Keywords:** *Compensation*, *head and neck*, *morbidity*, *operative time*, *otolaryngology*, *reimbursement*, *relative value unit*, *reoperation*

## Abstract

**Background:** The determination of accurate measures of evaluating surgeon work for reimbursement is poorly characterized. This study defines the correlation of surgical work relative value units (work RVUs) with several surrogate objective measures for otolaryngologic work. The defined surrogate objective measures evaluated in this study are length of hospital stay (LOS), operative time, 30-day mortality, 30-day unplanned readmission, 30-day reoperation, and 30-day morbidity.

**Methods:** We collected data on otolaryngologic cases from 2016 to 2018 from the American College of Surgeons National Surgical Quality Improvement Program. Pearson correlation coefficient was used to associate work RVUs with objective measures of surgeon work. Linear regressions were used to identify predictors of work RVUs from the surrogate objective measures. Studentized residuals were used to identify outlying procedures.

**Results:** Work RVUs correlated strongly with operative time (*R*=0.6775), 30-day readmission (*R*=0.6100), and LOS (*R*=0.6083); moderately with 30-day reoperation (*R*=0.5257) and 30-day morbidity (*R*=0.4842); and very weakly with 30-day mortality (*R*=0.1383). The best predictors for work RVUs based on multivariable linear regression analysis were morbidity, reoperation, and operative time. Analysis revealed that the projected work RVU is 12.23 units higher than the current value for excision of bone, mandible (Current Procedural Terminology [CPT] code 21025) and 19.48 units lower than the current value for resection/excision of lesion infratemporal fossa space apex extradural (CPT code 61605).

**Conclusion:** Using objective surrogate measures for time and intensity of physician work in head and neck cases may improve work RVU assignment accuracy compared to the current system of physician survey. Future investigation with additional objective parameters may be beneficial to make work RVU assignments less subjective.

## INTRODUCTION

Concerns about the rising costs of Medicare expenditures and low reimbursement rates for primary care physicians became prevalent in late 1980.^1^ This conversation prompted researchers to create the resource-based relative value scale (RBRVS) for physician reimbursement.^1^ The RBRVS was based on the conditions of a physician's time or work associated with a service, the cost of running a practice, and the opportunity cost of physician training payback during the course of their career.^1^ The predecessor of the Centers for Medicare and Medicaid Services (CMS), the Health Care Financing Administration, first implemented relative value units (RVUs) as a standard unit to measure physician work in 1992 to define reimbursement rates, a move that was supported by researchers in the years prior.^1-3^ With this implementation, the American Medical Association (AMA), which controls the Current Procedural Terminology (CPT) codes that are linked to the RBRVS, established the Relative Value Scale Update Committee (RUC) to provide a reliable bridge between practicing physicians and CMS.^1,4^ The RUC consists of professional members of the AMA and representatives from national societies of various medical specialties. The RUC generally meets thrice yearly to provide recommendations to CMS about the relative value of work so CMS can annually reevaluate its Medicare RBRVS and Physician Fee Schedule.^4^

Work RVU, a measure of surgeon work, has 3 building blocks: (1) preservice (eg, reviewing records, case discussion, and preparing for surgery); (2) intraservice (eg, intraoperative period from first incision to closing the incision); and (3) postservice (eg, global surgical period including recovery room time and inpatient hospital stay).^5^ Notably, work RVU calculation for primary care providers may include different variables. For surgeons and physicians, however, the time and intensity associated with their respective building blocks are reflected in the final work RVU assignment for a service. The RUC obtains these estimations from physician surveys conducted by specialty societies. To account for potential bias or overestimations of operative time from survey responses, the RUC generally uses the operative time that falls in the 25th percentile of survey responses. Requests for reevaluation are a tool that specialty societies may use to adjust the RVU of a specific procedure. In instances of reevaluation requests, societies often feel work RVUs are undervalued because of factors such as advances in technology and updates in patient risk profiles. On the other hand, the RUC reserves the right to deny reevaluation requests.^4,6^

Currently, no database exists that can accurately measure time and intensity for all 3 building blocks so that work RVU assignments can be verified. However, we believe that surrogate objective measures for surgeon work (ie, the 3 building blocks) should be evaluated to provide evidence for updating work RVUs and claiming appropriate surgical compensation. In fact, studies in different surgical specialties found that length of hospital stay (LOS), morbidity, mortality, reoperation, unplanned readmissions, and operative time correlate with work RVUs and should be used in work RVU reevaluation.^5,7^ Therefore, the objective of this study was to define the correlation of work RVUs with surgeon work in otolaryngology using the aforementioned measures. The database used, the American College of Surgeons National Surgical Quality Improvement Program (NSQIP), only includes major inpatient and outpatient otolaryngology cases; thus, the procedures evaluated in this study are limited to such.

## METHODS

We combined the NSQIP data from 2016 to 2018 for analysis in this study. The operative time for each CPT code in concurrent cases is inseparable and likely results in multiple RVUs being billed for cases with multiple surgeons. Therefore, we excluded any encounter involving more than one surgeon and/or having concurrent CPT codes. We only analyzed cases performed by otolaryngologists. We also eliminated any CPT code that had fewer than 25 associated encounters.

From the CPT codes selected, we collected data on the following variables: LOS, operative time, 30-day mortality, 30-day unplanned readmission, 30-day reoperation, and 30-day morbidity. Morbidity is defined as any serious adverse events that occurred within 30 days of the procedure, including surgical site infection, wound disruption, pneumonia, unplanned intubation, pulmonary embolism, acute renal failure, cardiovascular accident or stroke, cardiac arrest, myocardial infarction, bleeding that required transfusion, and septic shock.

Median values were used for analyses of LOS and operative time. Means of percentages were used for analyses in 30-day mortality, 30-day unplanned readmission, 30-day reoperation, and 30-day morbidity.

SAS software, version 9.4 (SAS Institute Inc) was used to perform statistical analyses. Bivariate and multivariable linear regressions were used to identify predictors of work RVUs from the above variables. In addition, correlations between each variable and work RVUs were calculated using Pearson correlation coefficient. We defined absolute *R* values as follows: very strong if *R* is between 0.8 and 1.0, strong if *R* is between 0.6 and 0.79, moderate if *R* is between 0.4 and 0.59, weak if *R* is between 0.2 and 0.39, and very weak if *R* is <0.19. *P* values <0.10 were considered significant.

Studentized residuals, a statistical method to detect outliers based on the mean square error with the investigated target excluded, were used to identify outlying work RVUs in the bivariable and multivariable linear regressions. Work RVUs with studentized residuals <–2 or >2 were considered outliers. Based on bivariate and multivariable linear regression analyses, projected work RVUs were calculated. The difference between projected work RVU and actual work RVU was determined.

This study was designated exempt by the institutional review board at Albany Medical Center.

## RESULTS

We identified 110,795 otolaryngologic cases in the years 2016, 2017, and 2018. We excluded 1,201 cases with concurrent CPT codes, 26,150 cases with a secondary procedure, and 2,488 cases with both concurrent CPT codes and a secondary procedure. From the remaining 80,956 cases, only 41,666 cases were otolaryngologic, with 53 CPT codes represented. Only 43 CPT codes had more than 25 patient encounters, for a total of 41,554 cases included in our final analysis. All 43 CPT codes were defined for head and neck surgeries.

The mean work RVU for the procedures analyzed was 16.32 ± 9.49, ranging from 1.56 for excision of tongue without closure (CPT code 41110) to 38.81 for total laryngectomy with radical neck dissection (CPT code 31365). Table 1 shows the 43 CPT codes with their associated work RVUs and procedural frequencies.

**Table 1.** Case Mix, Frequency, Work Relative Value Unit (RVU), Operative Time, Length of Stay (LOS), Morbidity, Readmission, Reoperation, and Mortality for 43 Representative Otolaryngology Procedures

CPT Code	Procedure	Frequency	Mean Work RVU	Median Operative Time, min	Median LOS, days	Mean Morbidity, %	Mean Readmission, %	Mean Reoperation, %	Mean Mortality, %
31365	Laryngectomy total with radical neck dissection	39	38.81	402	9	15.38	12.82	7.69	0
15756	Free muscle/myocutaneous flap with microvascular anastomosis	29	36.94	502	8	34.48	13.79	20.69	0
61605	Resection or excision of neoplastic, vascular, or infectious lesion of infratemporal fossa, petrous apex; extradural	31	32.57	102	1	6.45	3.23	3.23	0
41135	Partial glossectomy with unilateral radical neck dissection	95	30.14	180	3	11.58	6.32	5.26	1.05
31360	Total laryngectomy without neck dissection	88	29.91	370	8	27.27	12.5	10.23	1.14
41150	Composite glossectomy with floor and mandibular resection	25	29.86	264	6	32	12	28	0
60254	Total/subtotal thyroidectomy with radical neck dissection	250	28.42	208	2	4	2	3.2	0.4
38724	Cervical lymphadenectomy with modified neck dissection	1,197	23.95	150	1	4.68	4.01	3.43	0.08
60270	Thyroidectomy with substernal split/transthoracic	57	23.2	130	1	5.26	3.51	1.75	0
60505	Parathyroidectomy/exploration of parathyroids with transthoracic echocardiogram	32	23.06	94	1	0	0	0	0
42426	Excision of parotid tumor/total parotid gland with unilateral radical neck dissection	100	22.66	184	2	6	3	3	0
60252	Total thyroidectomy/subtotal limited neck dissection	1,235	22.01	131	1	2.83	2.75	1.46	0.24
38720	Cervical lymphadenectomy	75	21.95	138	1	8	9.33	5.33	2.67
60502	Parathyroidectomy/exploration of parathyroids, re-exploration	63	21.15	94	0	3.17	3.17	3.17	0
42420	Excision of parotid tumor/total parotid gland dissection and facial nerve preservation	816	19.53	143	1	3.19	1.35	0.98	0.12
60260	Removal of remaining thyroidectomy tissue	895	18.26	80	1	1.9	2.01	0.78	0
60271	Thyroidectomy substernal, cervical approach	603	17.62	124	1	1.33	2.16	1.66	0.33
42415	Excision of parotid tumor/lateral parotid gland dissection and facial nerve preservation	2,379	17.16	127	1	2.52	1.35	0.97	0.04
60212	Partial unilateral thyroid lobectomy with contralateral lobectomy	83	16.43	98	1	2.41	1.2	0	0
41130	Glossectomy: hemiglossectomy	71	15.74	71	1	12.68	4.23	8.45	1.41
60500	Parathyroidectomy/exploration of parathyroids	2,741	15.6	85	0	1.42	2.12	0.62	0.07
60240	Total thyroidectomy	6,699	15.04	121	1	1.81	2.27	1.13	0.15
60225	Total unilateral thyroid lobectomy with contralateral lobectomy	317	14.79	91	1	0.32	1.58	0.32	0
42425	Excision of parotid tumor/total gland en bloc removal	44	13.42	131	1	4.55	2.27	6.82	0
38700	Suprahyoid lymphadenectomy	55	12.81	104	1	0	0	0	0
21044	Excision of malignant tumor, mandible	29	12.8	363	7	31.03	3.45	3.45	0
42842	Radical resection of tonsil without closure	69	12.23	124	2	1.45	7.25	4.35	0
42120	Resection of palate/extensive resection of lesion	66	11.86	57.5	0	4.55	1.52	0	1.52
60210	Partial unilateral thyroid lobectomy with or without isthmusectomy	1,395	11.23	88	1	2.22	1.36	1.29	0.07
60220	Total unilateral thyroid lobectomy with or without isthmusectomy	6,473	11.19	84	1	1.14	1.07	0.82	0.03
41120	Glossectomy: <½ tongue	447	11.14	49	0	2.68	1.57	2.46	0
21025	Excision of bone, mandible	83	10.03	355	6	26.51	3.61	7.23	1.2
60200	Excision of cyst/thyroid adenoma/isthmus transection	134	10.02	65	0	2.24	2.99	0.75	0.75
42410	Excision of parotid tumor/lateral lobe of parotid gland without nerve dissection	623	9.57	95	0	2.73	0.64	0.8	0
42950	Pharyngoplasty	38	8.27	39.5	0	0	2.63	0	0
38542	Deep jugular node dissection	58	7.95	54	0	0	3.45	1.72	0
42440	Excision of submandibular, submaxillary glands	961	6.14	74	0	3.02	1.46	0.73	0.1
42870	Excision/destruction of lingual tonsil	63	5.52	49	1	4.76	3.17	3.17	0
42826	Tonsillectomy: ½, age >12	12,410	3.45	20	0	1.24	2.57	3.74	0.02
41112	Excision of tongue lesion with closure of anterior two-thirds	319	2.83	21	0	0.31	1.88	0.31	0
41116	Excision of floor of mouth lesion	108	2.52	38	0	2.78	4.63	0.93	0
42808	Excision/destruction of pharyngeal lesion	118	2.35	15.5	0	2.54	2.54	0.85	0
41110	Excision of tongue lesion without closure	141	1.56	27	0	0.71	0.71	0.71	0
N/A	All procedures evaluated	41,554	16.32 ± 9.49	133.55 ± 111.29	1.67 ± 2.43	6.58 ± 9.34	3.62 ± 3.46	3.52 ± 5.35	0.26 ± 0.56

CPT, Current Procedural Terminology.

The mean of the median operative times of 43 CPT codes was 133.55 minutes ± 111.29 minutes. The mean of the median LOS was 1.67 days ± 2.43 days. The means of morbidity percentage, readmission percentage, reoperation percentage, and mortality percentage were 6.58% ± 9.34%, 3.62% ± 3.46%, 3.52% ± 5.35%, and 0.26% ± 0.56%, respectively. Strong correlations were observed between work RVU and operative time (*R*=0.6775), 30-day readmission (*R*=0.6100), and LOS (*R*=0.6083). Moderate correlations were observed between work RVU and 30-day reoperation (*R*=0.5257) and 30-day morbidity (*R*=0.4842). However, a very weak correlation was observed between work RVU and 30-day mortality (*R*=0.1383). Bivariable linear regression demonstrated that the variables operative time, LOS, reoperation and readmission within 30 days, and 30-day morbidity were predictors of work RVU. Mortality within 30 days was not predictive of work RVU (Figure).

**Figure.**
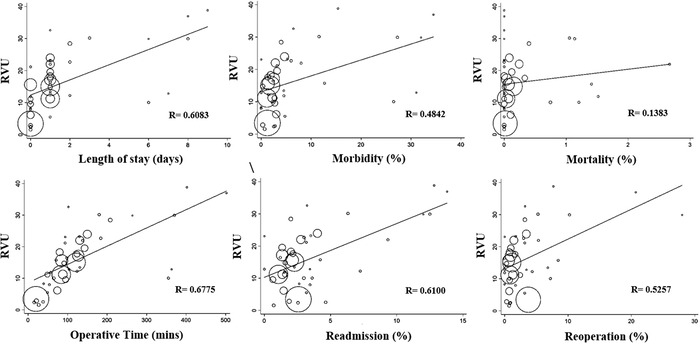
Correlation between the 6 surrogate objective measures of time and intensity of 43 surgical procedures and work relative value unit (RVU).

Every minute increase in operative time yields a work RVU increase of 0.0577 units (95% CI 0.0380 to 0.0775; *P*<0.001). A 1-day increase in LOS leads to an increase of 2.368 units in work RVU (95% CI 1.393 to 3.342; *P*<0.001). Similarly, a 1% increase in morbidity, readmission, and reoperation leads to an increase of 0.4917 units (95% CI 0.2115 to 0.7720; *P*<0.001), 1.673 units (95% CI 0.9877 to 2.3589; *P*<0.001), and 0.9321 units (95% CI 0.4564 to 1.4079; *P*<0.001) in work RVU, respectively. No linear correlation was observed between mortality and work RVU (95% CI –2.9493 to 7.6331; *P*=0.377) (Figure).

The predictors for work RVU based on multivariable linear regression analysis of the other 5 variables were morbidity (*P*<0.005), operative time (*P*<0.001), and reoperation (*P*=0.085). Readmission (*P*=0.244) and LOS (*P*=0.367) were not strongly associated with work RVUs using multivariable linear regression analysis. Using a significance threshold of <0.1, multivariable linear regression analysis was repeated with only morbidity, operative time, and reoperation. All 3 variables were statistically significant in this model: morbidity (–0.9634, 95% CI –1.5076 to –0.4193; *P*<0.001), operative time (0.0973, 95% CI 0.0619 to 0.1327; *P*<0.001), and reoperation (0.9701, 95% CI 0.3434 to 1.5968; *P*=0.003). The *R^2^* of this model was 0.6018.

Table 2 shows the outliers of the bivariable linear regression and the multivariable linear regression models and the difference between the predicted and actual work RVUs. Excision of bone, mandible (CPT code 21025), work RVU of 10.03 units, was an outlier in multiple models, including the bivariable linear regression of operative time, LOS, and morbidity. Multivariable linear regression analysis of reoperation, morbidity, and operative time also found excision of bone, mandible to be an outlier, with a projected work RVU of 22.26 units, 12.23 units higher than the current work RVU.

**Table 2.** Outlier Procedures Based on Univariable and Multivariable Linear Regressions With Projected Work Relative Value Unit (RVU)

Variable	CPT Code	Procedure	Studentized Residuals	Actual Work RVU	Projected RVU	Difference
Operative time	21025	Excision of bone, mandible	–3.1790	10.03	29.11	19.08
	21044	Excision of malignant tumor, mandible	–2.7302	12.8	29.57	16.77
	61605	Resection/excision of lesion infratemporal fossa space apex extradural	+2.7997	32.57	14.50	–18.07
Readmission	41116	Excision of floor of mouth lesion	–2.1523	2.52	18.02	15.5
	60254	Total/subtotal thyroidectomy with radical neck dissection	+2.0495	28.42	13.62	–14.8
	61605	Resection/excision of lesion infratemporal fossa space apex extradural	+2.3715	32.57	15.67	–16.9
Length of stay	21025	Excision of bone, mandible	–2.4148	10.03	26.56	16.53
	21044	Excision of malignant tumor, mandible	–2.4073	12.8	28.93	16.13
	61605	Resection/excision of lesion infratemporal fossa space apex extradural	+2.5220	32.57	14.72	–17.85
Reoperation	31365	Laryngectomy total with radical neck dissection	+2.4609	38.81	20.20	–18.61
	61605	Resection/excision of lesion infratemporal fossa space apex extradural	+2.1344	32.57	16.04	–16.53
Morbidity	21025	Excision of bone, mandible	–2.1423	10.03	26.11	16.08
	21044	Excision of malignant tumor, mandible	–2.1382	12.8	28.34	15.54
	31365	Laryngectomy total with radical neck dissection	+2.3279	38.81	20.64	–18.17
	61605	Resection/excision of lesion infratemporal fossa space apex extradural	+2.0395	32.57	16.25	–16.32
Reoperation, morbidity, operative time	21025	Excision of bone, mandible	–2.3344	10.03	22.26	12.23
	41135	Partial glossectomy with unilateral radical neck dissection	+2.1208	30.14	17.71	–12.43
	61605	Resection/excision of lesion infratemporal fossa space apex extradural	+3.6558	32.57	13.09	–19.48

Notes: Morbidity is defined as any serious adverse events that occurred within 30 days of the procedure. Reoperation is defined as return to the operating room within 30 days of the procedure. Operative time is defined as time from skin incision to skin closure of the procedure in minutes. Mortality is not included as no linear correlation was found between mortality and work relative value units (95% CI –2.9493 to 7.6331; *P*=0.377).

CPT, Current Procedural Terminology.

Similarly, resection/excision of lesion infratemporal fossa space apex extradural (CPT code 61605), work RVU of 32.57 units, was an outlier in operative time, readmission, LOS, reoperation, and morbidity, as well as an outlier in the multivariable regression analysis of reoperation, morbidity, and operative time (19.48 units lower than the current work RVU).

Studentized residuals for the 43 different CPT codes based on univariable and multivariable linear regression analysis are shown in Table 3.

**Table 3.** Studentized Residuals for 43 Current Procedural Terminology (CPT) Codes Based on Univariable and Multivariable Linear Regression Analysis

CPT Code	Procedure	Reoperation, Morbidity, Operative Time	Operative Time	LOS	Morbidity	Readmission	Reoperation
31365	Laryngectomy total with radical neck dissection	0.1507	1.0828	0.7698	2.3279	1.0373	2.4609
15756	Free muscle/myocutaneous flap with microvascular anastomosis	–0.9991	–0.1089	0.8162	0.9385	0.5329	0.6567
61605	Resection or excision of neoplastic, vascular, or infectious lesion of infratemporal fossa, petrous apex; extradural	3.6558	2.7997	2.5220	2.0395	2.3715	2.1345
41135	Partial glossectomy with unilateral radical neck dissection	2.1208	1.6312	1.4419	1.3891	1.2551	1.5381
31360	Total laryngectomy without neck dissection	0.6995	–0.0097	–0.1989	0.4345	–0.1829	0.92581
41150	Composite glossectomy with floor and mandibular resection	0.4333	0.8729	0.4514	0.1373	–0.0693	–1.6773
60254	Total/subtotal thyroidectomy with radical neck dissection	0.4528	1.1276	1.5289	1.6453	2.0495	1.5632
38724	Cervical lymphadenectomy with modified neck dissection	0.6989	0.9565	1.2341	1.0334	0.9255	0.9556
60270	Thyroidectomy with substernal split/transthoracic	1.2611	1.0157	1.1304	0.9053	0.9374	1.0596
60505	Parathyroidectomy/exploration of parathyroids with transthoracic echocardiogram	1.2699	1.3062	1.1112	1.2163	1.7682	1.2573
42426	Excision of parotid tumor/total parotid gland with unilateral radical neck dissection	0.2240	0.4877	0.7356	0.7942	0.9801	0.8430
60252	Total thyroidectomy/subtotal limited neck dissection	0.7056	0.8334	0.9678	0.9074	0.9482	0.9440
38720	Cervical lymphadenectomy	0.7803	0.7661	0.9596	0.5899	–0.5371	0.4846
60502	Parathyroidectomy/exploration of parathyroids, re-exploration	0.9355	1.0216	1.1802	0.7814	0.7365	0.6341
42420	Excision of parotid tumor/total parotid gland dissection and facial nerve preservation	0.2418	0.3779	0.6343	0.5843	0.9350	0.6888
60260	Removal of remaining thyroidectomy tissue	0.8632	0.7190	0.4657	0.5082	0.6120	0.5540
60271	Thyroidectomy substernal, cervical approach	–0.1673	0.2625	0.3811	0.4655	0.4942	0.3731
42415	Excision of parotid tumor/lateral parotid gland dissection and facial nerve preservation	0.0072	0.1776	0.3205	0.3390	0.6156	0.3963
60212	Partial unilateral thyroid lobectomy with contralateral lobectomy	0.4810	0.3070	0.2244	0.2583	0.5497	0.4185
41130	Glossectomy: hemiglossectomy	1.1618	0.4320	0.1337	–0.4283	–0.2103	–0.6427
60500	Parathyroidectomy/exploration of parathyroids	0.2989	0.2961	0.4291	0.2174	0.2358	0.24418
60240	Total thyroidectomy	–0.3809	–0.0783	0.0418	0.1279	0.1282	0.1163
60225	Total unilateral thyroid lobectomy with contralateral lobectomy	–0.0512	0.1317	0.0090	0.1860	0.2484	0.17964
42425	Excision of parotid tumor/total gland en bloc removal	–1.3202	–0.3900	–0.1709	–0.2258	–0.0858	–0.7389
38700	Suprahyoid lymphadenectomy	–0.5817	–0.2554	–0.2510	–0.0324	0.3387	–0.0277
21044	Excision of malignant tumor, mandible	–0.5154	–2.7302	–2.4073	–2.1382	–0.4264	–0.4229
42842	Radical resection of tonsil without closure	–1.5175	–0.5021	–0.6406	–0.1867	–1.3855	–0.5971
42120	Resection of palate/extensive resection of lesion	0.7299	–0.0094	–0.0651	–0.4122	–0.1247	–0.1445
60210	Partial unilateral thyroid lobectomy with or without isthmusectomy	–0.4351	–0.3490	–0.4595	–0.3516	–0.1741	–0.3694
60220	Total unilateral thyroid lobectomy with or without isthmusectomy	–0.4738	–0.3220	–0.4648	–0.2933	–0.1141	–0.3205
41120	Glossectomy: <½ tongue	0.0525	–0.0421	–0.1601	–0.3894	–0.2310	–0.5144
21025	Excision of bone, mandible	–2.3344	–3.1790	–2.4148	–2.1424	–0.8330	–1.2213
60200	Excision of cyst/thyroid adenoma/isthmus transection	–0.1813	–0.3330	–0.3081	–0.4979	–0.6932	–0.4567
42410	Excision of parotid tumor/lateral lobe of parotid gland without nerve dissection	–0.6606	–0.6442	–0.3678	–0.5808	–0.2352	–0.5188
42950	Pharyngoplasty	–0.2959	–0.3742	–0.5406	–0.5781	–0.8494	–0.5886
38542	Deep jugular node dissection	–0.8552	–0.5394	–0.5833	–0.6170	–1.0779	–0.8269
42440	Excision of submandibular, submaxillary glands	–0.8339	–0.9682	–0.8265	–1.0168	–0.8749	–0.9400
42870	Excision/destruction of lingual tonsil	–0.6564	–0.8506	–1.2303	–1.1995	–1.3516	–1.3087
42826	Tonsillectomy: ½, age >12	–1.2038	–0.9139	–1.1951	–1.2466	–1.5030	–1.6529
41112	Excision of tongue lesion with closure of anterior two-thirds	–0.9014	–1.0143	–1.2816	–1.2694	–1.4300	–1.3177
41116	Excision of floor of mouth lesion	–0.9341	–1.2036	–1.3251	–1.4590	–2.1523	–1.4314
42808	Excision/destruction of pharyngeal lesion	–0.6285	–1.0398	–1.3491	–1.4663	–1.6547	–1.4445
41110	Excision of tongue lesion without closure	–1.2159	–1.2555	–1.4611	–1.4559	–1.3403	–1.5330

LOS, length of stay.

## DISCUSSION

Of the 3 major considerations that account for the calculation of RVUs (physician work, the cost of running a practice, and opportunity cost of physician training), surgeon work contributes 50.9% of the total calculation. To further understand surgeon work, it is important to note that the following variables are all included in calculating surgeon reimbursement: time to complete a service, technical skill and physical labor, cerebral effort and decision-making, and risk to the patient.^4^ Work RVUs are intended to encompass the following distinct components: preoperative assessment, operative time and effort, and inpatient postoperative management.^8^ As explained earlier, the RUC currently obtains work RVU estimates from physician surveys conducted by each respective specialty society. However, we believe that surrogate measures for time and intensity of procedures can be used so that work RVU assignments can be verified. Studies in different surgical specialties found that LOS, morbidity, mortality, reoperation, unplanned readmissions, and operative time are correlated with work RVUs and should be used in work RVU reevaluation.^5,7^ In 2007, Smith et al explained the effort of the Society of Thoracic Surgeons to use objective data such as operative time and LOS from the society database, conduct national surveys to estimate work intensity, and work with the RUC and CMS to achieve more equitable work RVU assignments for their cases.^9^ In fact, the mean work RVU in otolaryngology was found to be 3.05 units lower compared to general surgery, while that of cardiothoracic surgery was 7.78 units higher than general surgery in a 2019 study.^5^ Therefore, the field of otolaryngology could likely benefit from investigating these objective measures. The RUC should be informed of any discovered undervaluation compared to our surgical counterparts and subsequently lobbied for more accurate reimbursement for surgeon work in otolaryngology.

In a study that determined the correlation of work RVU with operative time, LOS, readmission, and reoperation of surgical procedures in various specialties, these 4 measures explained 80% of the variation in work RVUs.^5^ Multivariable linear regression analysis in our study revealed that only morbidity, operative time, and reoperation are statistically significant variables in the determination of work RVU and explained only 60.18% of work RVU calculation (the *R^2^* of the multivariable linear regression was 0.6018). The lack of statistical significance among other studied variables implies that work RVUs in otolaryngology, specifically head and neck surgery, are poorly correlated with studied objective measures. Our group believes that increased utilization of objective measures from available databases could improve the accuracy of work RVU assignment during updating processes.

Morbidity, an indicator for postoperative management work and risk to patients, was a negative predictor of work RVU by multivariable regression. All 43 CPT codes explored in our study were head and neck surgeries, and patients undergoing head and neck procedures typically have multiple medical comorbidities.^10^ This negative correlation leading to an undervaluation of work RVUs could be attributable to the lack of accounting for medical complexity and complications related to comorbidities that surgeons manage postoperatively. Similarly, LOS and readmission were not predictive of work RVUs by multivariable regression. The lack of association of work RVUs to LOS and readmission is concerning, as head and neck cancer cases are often linked to prolonged postoperative courses with high complication rates. In the literature, the average LOS and the readmission rate in head and neck cancer diagnoses were reported to be 6.6 days and 13.8%, respectively.^11,12^ Therefore, morbidity, LOS, and readmission rate should be thoroughly incorporated in assigning work RVUs to avoid undercompensation for head and neck cancer surgeons.

Mortality was very weakly correlated to work RVUs. Multiple studies have corroborated the effect of mortality on work RVUs across surgical specialties.^5,7,13-15^ Because mortality rates associated with the procedures included in this study were so low, precisely assessing the association between work RVUs and mortality may not be possible. However, like our study, various studies also describe the poor relationship between mortality and case complexity with work RVUs.^5,7,13-16^ The reasons for this poor correlation are most likely multifactorial and confounded by patient-related comorbidities and the health care resources, or lack thereof, available to them upon discharge.^7,16^

In addition, our study identified various outlying procedures. For example, excision of bone mandible (CPT code 21025) was found to be undervalued in multiple analyses, meaning surgeons were not properly compensated for completing the procedure. This difference could result from the procedure being improperly evaluated, leading to an erroneous work RVU assignment. However, the actual operative time was long (355 minutes). This finding raises the possibility that the correct CPT code is not being selected when billing for this procedure. The literature documents that CPT coding errors are prevalent across various medical specialties.^17,18^ Conceivably, with regard to excision of bone mandible (CPT code 21025), the following similar but more complex CPT codes were not selected: 21040 (excision of benign tumor or cyst of mandible by enucleation and/or curettage) with a work RVU of 15.11; 21046 (excision of benign tumor or cyst of mandible requiring intra-oral osteotomy [eg, locally aggressive or destructive lesion]), with a work RVU of 32.44; or 21045 (excision of malignant tumor of mandible, radical resection), with a work RVU of 35.41. This point affirms the need for surgeons to take ownership of accurate codes and selection of codes to avoid an undervaluation of procedures. The projected work RVU for excision of bone, mandible resulting from our study's multivariable analysis is 22.26 units, 12.23 units higher than the actual work RVU; therefore, physicians and surgeons must be careful to select the code that best fits their procedure for proper compensation. A similar finding of undervalued work RVU was seen in CPT code 21044 (excision of malignant tumor, mandible), which has a median operative time of 363 minutes and a work RVU of 12.8 units. CPT code descriptions could also be updated to make them clearer for intended use. The long operative times of these 2 procedures call into question whether the procedures included reconstruction. We avoided this type of error by selecting cases for which only a single CPT code was billed.

An example of an overcompensated procedure is resection/excision of lesion infratemporal fossa space apex extradural (CPT code 61605). This procedure had a lower operative time and fewer complications than predicted. Given a deeper look, perhaps this procedure is compensated for the anatomically difficult locations surrounded by major structures that induce a potential for elevated complications. These complications may be minimized by the expertise and extensive training completed by the surgeons who perform these operations. Therefore, considering the possible risk to patients weighing on the surgeon and the opportunity cost payoff for training time, this procedure may be properly reimbursed. A future direction of this study could be to analyze the cost-effectiveness associated with the time, money, and effort surgeons spend in subspecialty training, as well as meeting continued medical education requirements to successfully perform high-risk procedures.

Although we believe that the results of our study are compelling and should be taken into consideration when assigning work RVUs to the studied procedures, we also acknowledge the limitations of our data. First, the NSQIP database only includes data from major cases from a sampling of institutions around the country. Together with our strict exclusion of cases with concurrent CPT codes and more than one surgeon, only 43 procedures were included, and all were head and neck procedures. Although the strict exclusion criteria made our results more readily ascribable to procedures involving a single CPT code, these criteria may limit the application of our findings to concurrent or multidisciplinary surgeries. Further studies may be warranted to explore how these objective measures could be used to reimburse for cases associated with multiple surgeons or CPT codes. For example, many head and neck surgeries require reconstruction; these procedures are likely to be more time consuming and to require multiple surgeons compared to surgeries that involve a single discipline and do not require reconstruction. Analysis of individual surgeon work associated with these complex and multidisciplinary procedures, therefore, is a potential outlet for future exploration. Our study only investigated 6 objective measures that could contribute to the time and intensity of the preservice, intraservice, and postservice building blocks of work RVUs. These variables do not represent the only factors that contribute to surgeon time and case complexity. Therefore, the data need to be interpreted with caution. Finally, because the NSQIP database represents a sampling of cases from a group of 703 large NSQIP hospitals, the data may not be generalizable among all institutions.^19^

## CONCLUSION

This study exposes incongruities between the variables studied in the NSQIP database and assigned work RVUs for head and neck procedures. Objective surrogate measures for surgeon work could improve work RVU assignment accuracy for the outlier procedures that are not accurately represented. Using such objective measures may be more reliable compared to the current system of work RVU assignment based on physician survey. Future investigation with additional objective parameters may be further beneficial to make work RVU assignment less subjective. Outlying work RVUs, therefore, should be reevaluated by the RUC for proper compensation based on objective measures of surgeon work.

## Notes

*This article meets the Accreditation Council for Graduate Medical Education and the American Board of Medical Specialties Maintenance of Certification competencies for Patient Care, Medical Knowledge, Systems-Based Practice, and Practice-Based Learning and Improvement.*
